# Chemical Characterization and Temporal Variability of Pasta Condiment By-Products for Sustainable Waste Management

**DOI:** 10.3390/foods13183018

**Published:** 2024-09-23

**Authors:** Lorenzo Strani, Giulia Farioli, Marina Cocchi, Caterina Durante, Alessandra Olarini, Samuele Pellacani

**Affiliations:** Department of Chemical and Geological Sciences, University of Modena and Reggio Emilia, Via Campi 103, 41125 Modena, Italy; lorenzo.strani@unimore.it (L.S.); 255751@studenti.unimore.it (G.F.); marina.cocchi@unimore.it (M.C.); alessandra.olarini@unimore.it (A.O.); samuele.pellacani@unimore.it (S.P.)

**Keywords:** spectroscopy, chemometrics, waste, by-product

## Abstract

Sustainable waste management is an extremely important issue due to its environmental, economic, and social impacts. Knowledge of the chemical composition of the waste produced is a starting point for its valorization. This research focuses, for the first time, on the by-products of pasta condiment production, starting with their characterization. In particular, the presence of potential bioactive compounds and their variability over time have been studied. The latter aspect is crucial for the subsequent valorization of these by-products. In addition to acidity and total phenolic content, an untargeted strategy was adopted, using spectroscopic and chromatographic techniques coupled with chemometrics, to study waste samples coming from four single condiment production lines, i.e., Genoese pesto, tomato, ricotta, and ragù sauces. The presence of lycopene, polyphenols, and several valuable volatile compounds was highlighted. Their presence and relative amounts vary mainly according to the presence of tomatoes in the sauce. The results obtained at different storage times (after 0, 7, 10, and 15 days) showed that the samples studied, despite having similar chemical characteristics, underwent changes after one week of storage and then presented a relatively stable chemical profile. A general decrease is observed after 7 days for almost all the chemical variables monitored, so careful planning within the first days is required to obtain a high recovery.

## 1. Introduction

Tomatoes, a globally significant crop, surpassed an annual production of 180 million tons in 2021, with Italy contributing 53% of the total European production, reaching 5.2 million tons in 2022 [1]. Despite the prominence of the tomato processing industry worldwide, disposal challenges persist due to substantial waste production, imposing environmental burdens [2]. Addressing these challenges aligns with the priorities outlined in the European Union’s Agenda 2030, particularly Target 12.3, which emphasizes reducing food waste and exploring strategies for requalification. During tomato processing, important by-products are produced, including peels, seeds, and pomace, representing a total of 3–5% (*w*/*w*) of the raw tomato [3]. Several studies have been undertaken to conduct a comprehensive chemical characterization of these by-products, with the aim of exploring potential avenues for their reuse [3,4,5,6,7]. Particularly, fibers constitute one of the main components, ranging from 16% in seeds to 78.56% in peels on a dry basis. Other compounds like oil and proteins exhibit variations depending on factors like variety, geographical location, and ripening stages. In particular, the content (on a dry basis) of protein could vary from 1.85% in peels to 40.94% in seeds, and oil ranges from 1.63% in peels to 24.50% in seeds [5].

Concerning tomato pomace, it comprises a substantial quantity of pulp and peels, ranging from 56% to 67% on a dry basis, with the remaining portion consisting of seeds [3,5]. It is an excellent source of antioxidants, including carotenoids, phenolic compounds, and ascorbic acid, which are widely recognized as having strong radical scavenging properties [5]. Indeed, carotenoids and polyphenols are among the tomato’s major bioactive compounds thanks to their antioxidant and anti-inflammatory properties [8,9] and play a crucial role in preventing chronic illnesses, mainly cancers or cardiovascular diseases [10,11]. In a recent study, Nour et al. [12] stated that dried tomato wastes contain considerable amounts of lycopene (510.6 mg/kg) and β-carotene (95.6 mg/kg) and exhibit good antioxidant properties. Other studies have found that 65% of tomato flavanols, including quercetin, can resist common tomato processing methods and thus are found in tomato-derived products, such as tomato juice or tomato puree, which are particularly rich in these compounds [13]. The health benefits from processed tomato products may be similar or even higher than those of raw tomatoes, making tomato-derived products a perfect alternative to functional food [13]. These findings are optimal premises to motivate studies aimed at recovering lycopene and polyphenols from tomato processing by-products, including tomato-based condiments.

However, significant waste is constituted not only by peel and seeds but also by the finished product itself, as different faults can occur during the production process, leading to non-compliant batches or products that do not meet established quality standards, e.g., faults may include fluctuations in the quality of raw materials, inaccuracies in ingredient measurement, incorrect temperatures, or inappropriate cooking times [14]. Moreover, errors in the operation of packaging machines can result in defective packaging or product losses, affecting the freshness and integrity of the condiment. The complexity increases when different tomato-based condiments are produced on the same production line, raising the risk of cross-contamination in the absence of adequate preventive precautions. All these production errors cause the main disposal problem for the tomato processing industry [15]. Despite the potential value of tomato-based by-products, current disposal methods, such as animal feed production and landfill dumping, underutilize their resource potential. There is a lack of studies focusing on the characterization of pasta condiment wastes, whereas its valorization would be crucial to mitigate the environmental impact of this agri-food industry while fostering new ways of economic development and circular economy.

The aim of this research was to characterize the chemical profile of pasta condiment by-product waste from pasta sauce production lines in order to assess the potential reservoir of high-added value compounds. To the best of our knowledge, this is the first time that pasta sauce waste has been studied. Therefore, in this study, different types of pasta condiment wastes, namely Genoese pesto, ragù sauce, ricotta pesto, and tomato sauce, were chemically characterized with a twofold objective: (i) to identify the presence of potential compounds of interest and (ii) to assess their stability over time, considering the time period needed to deliver the by-product to the eventual processing industry In particular, it must be stressed that knowledge of this temporal dimension is essential to elucidate potential alterations in the chemical profiles of these by-products. As these could serve as precursors for any intended application, according to the principles of circular economy, it is essential to know their stability in time to assess the correct recovery for further feasibility analysis and economic evaluation.

Therefore, in the present study, waste samples coming from four different condiments production lines, in which tomatoes coexist with various other ingredients such as cheese, meat, and basil, were chemically characterized determining bulk factors such as pH and total polyphenol content, the volatile fraction sampled by solid-phase microextraction and analyzed by gas chromatography-mass spectrometry (SPME-GC-MS), as well as by spectroscopic techniques, namely ultraviolet-visible (UV–Vis) and near-infrared (NIR). The stability of the chemical composition of the studied by-products was investigated at 0, 7, 10, and 15 days of storage. These storage times were chosen as representative of conditions met during production and marketing.

pH and polyphenols are the most important bulk factors that define the taste and the first impression of a tomato or tomato processed products, together with the aroma [16,17], which is profiled by the volatile fraction and may vary significantly according to storage time [17].

To speed/optimize the analysis of the SPME-GC-MS responses and to maximize the information extraction in terms of resolved peaks it has been used the PARADISe method [18], which is a recently developed chemometric tool allowing both deconvolution of overlapped peak and putative compounds identification by interface with the NIST library. Several studies have shown its ability to facilitate the efficient extraction of deconvoluted peak areas and the potential identification of key compounds within complex datasets [19].

NIR and UV–Vis spectroscopies were considered to characterize specific compounds, e.g., lycopene with UV–Vis, but mostly as fast, low-cost fingerprinting techniques to obtain a first overview of the different kinds of waste by-products analyzed since the aim of this study was not only to elucidate the inherent complexities of the chemical profiles but also to discern significant trends and variations within the data, especially during the studied storage periods. To this aim, principal component analysis (PCA) [20] was applied to the NIR and UV–Vis spectra.

## 2. Materials and Methods

### 2.1. Chemicals

Sodium hydroxide standard solution (0.1 M NaOH), gallic acid standard, Folin–Ciocalteu reagent, diethyl ether, petroleum ether, and n-hexane (purity 99%), anhydrous sodium sulfate and potassium hydroxide were supplied by Carlo Erba (Milan, Italy). Standards of 2-phenylethyl acetate and 4-methyl-2-pentanol were purchased, respectively, from Merck Life Science (Milan, Italy) and Honeywell Fluka (Charlotte, NC, USA). Methanol, ethanol, and acetone, all of HPLC grade, were provided by Sigma Aldrich (Milan, Italy). Deionized water (ASTM D1193-91 type I [21], with a resistivity of 18.2 MΩ cm^−1^, ASTMD international, West Conshohocken, PA, USA) for samples, standards, and mobile phase preparation was obtained by a Millipore Milli-Q 185 Plus system.

### 2.2. Samples and Storage Conditions

The waste samples analyzed in this study, hereafter indicated as “sauce”, were kindly provided by an Italian company (anonymous for privacy), and they came from the following production batches: (i) tomato, (ii) ragù with meet, (iii) Genovese pesto and (iv) ricotta and walnut pesto condiments. These wastes should have a composition very similar to that of the starting sauces, as they are basically the result of errors during the production process (as previously explained). Below are details of the ingredients that should be present in the respective seasoning sauces.

(i) Tomato sauce: tomato pulp 72%, tomato concentrate 4%, onions, vegetable oils (olive, sunflower), basil 2%, sugar, salt, natural flavors.

(ii) Ragù with meat: pork meat 25%, water, tomato pulp 18%, tomato concentrate 16.5%, red wine 6%, carrots 4%, onions, corn starch, vegetable oils (olive, sunflower), sugar, salt, flavors, rosemary, garlic.

(iii) Genoese pesto: vegetable oils (olive, sunflower), fresh basil 30%, cashew nuts, Parmigiano Reggiano PDO 5% (milk), corn fiber, whey powder, salt, whey proteins, extra virgin olive oil, sugar, basil extract, natural flavors (milk), acidity regulator (lactic acid, garlic).

(iv) Pesto with ricotta and walnuts, hereafter called ricotta pesto: vegetable oils (olive, sunflower), ricotta 20% (whey powder, milk), tomato pulp 17,3%, glucose syrup, tomato concentrate 5.7%, walnuts 5%, basil 30%, Grana Padano PDO 5% (milk, egg derivative: lysozyme), salt, cashew nuts, whey powder, sugar, rice starch, acidity regulator: lactic acid, garlic, natural flavor (milk), buttermilk powder (milk).

All the samples were stored at 4 °C and analyzed at 0 (i.e., as soon as the sample was collected), 7, 10, and 15 days of storage.

### 2.3. Determination of pH

Determination of pH was carried out using a pH 510 pH meter (XS INSTRUMENTS, Carpi, MO, Italy), previously calibrated with buffer solutions at pH 7.0 and 4.0.

pH measurements were carried out in triplicate.

### 2.4. Determination of Total Phenolic Content

Determination of the total phenolic content was carried out following Folin–Ciocalteu method [22]. Briefly, 0.3 g of dried waste sauce was transferred into a 100 mL round-bottom flask, and 20 mL of an 80% methanol solution was added to the sample. The phenolic component extraction occurred under reflux on a water bath at 80 °C for 30 min. The extract was filtered through a #202 filter paper, and the residue underwent a second extraction. The two filtered solutions were combined in a 50 mL volumetric flask and diluted to 50 mL with an 80% methanol solution. 0.5 mL of prepared extract was mixed with 5.0 mL of Folin–Ciocalteu reagent, previously diluted 1:10 with distilled water. After a 4 min reaction, 4.0 mL of a 7.5% sodium carbonate solution was added and mixed. After incubation at room temperature for 30 min, absorbance was measured at 750 nm using an Ultrospec 3000 UV spectrophotometer (Pharmacia Biotech, Uppsala, Sweden). All the processes mentioned above for the extracts were applied for the different concentrations (0.02–0.2 mg/mL) of gallic acid solution as a standard to prepare a calibration curve. Total phenolic content was expressed in grams of gallic acid per 100 g of dried sauce (g gallic acid/100 g dried sauce) [22]. All the measurements were carried out in triplicate.

### 2.5. Qualitative Determination of Lycopene

In the present study, a qualitative determination of lycopene was exclusively conducted as it represents an exploratory investigation on the chemical characterization of the investigated waste pasta condiments. Further researchers, built upon the insights achieved from this preliminary investigation, will be dedicated to quantitative assessment of lycopene.

Five grams of sample was introduced into a 250 mL flask, followed by the addition of 25 mL of a 2:1:1 n-hexane:methanol:acetone mixture. All glassware in the laboratory was wrapped with aluminum foil to prevent degradation of lycopene in light [7]. After 30 min of stirring, the supernatant of the organic phase was filtered and transferred to a separatory funnel. The extraction procedure was repeated five times on the same sauce sample to maximize the recovery of lycopene present in the samples. The entire organic phase extracted from each sample was subsequently transferred to a 50.00 mL volumetric flask and diluted with n-hexane. Subsequently, 5.00 mL of this solution was further diluted to 10.00 mL in a volumetric flask with n-hexane. UV–Vis Spectra were collected by V-770 UV–Visible/NIR spectrophotometer (Jasco Europe, Milan, Italy) equipped with a PMT detector for the UV to visible region. The spectra were recorded in absorbance mode, ranging from 350 to 800 nm, with a bandwidth of 1.0 nm and a scan rate of 400 nm/min.

### 2.6. Near-Infrared Analysis

NIR spectra were acquired by dispensing approximately 5 g of sample into a sample holder (plastic vessel) with almost the same thickness (less than 4 mm) to avoid any scattering in the acquisition. The NIR spectra were obtained in 9 replicates.

The NIR spectra were collected using a portable NIR spectrometer, poliSPEC NIRe (ITPhotonics S.r.l., Fara Vicentino, Italy). Due to its diffraction grating and the double chip InGaAs 512 pixels sensor with a controlled cooling system, poliSPEC NIRe covers the spectral range of 930–1700 nm with an average numerical resolution of 3.2 nm and an average optical resolution at the half width at half maximum (HWHM) of 3.25 nm.

### 2.7. Determination of the Volatile Fraction by SPME-GC-MS

SPME-headspace analysis was carried out with a DVB/CAR/PDMS fiber (50/30 μm × 10 mm, diameter of 80 μm, Restek Corporation, Bellefonte, PA, USA), attached to an SPME fiber holder (Supelco, Bellefonte, PA, USA) for the extraction procedure. Around 1.0 g of sample was transferred into a 20 mL glass vial with an aluminum crip top closure and blue-silicone/PTFE septum. A 50 μL amount of 4-methyl-2-pentanol standard solution (100 mg/L) and 50 μL of 2-phenylethyl acetate standard solution (100 mg/L) were used as internal standards. All the areas were normalized to the standard one. All the sauce samples were stored at 4 °C and analyzed at 0 (i.e., as soon as the sample was collected), 7, 10, and 15 days of storage, for a total of 16 samples. The sample was incubated at 50 °C for 30 min, and then SPME fiber was exposed for 35 min [23]. Then, the fiber was manually transferred to the split/splitless injector of the Agilent 7890B GC System coupled with an Agilent HP 5973 mass spectrometer (Agilent Technologies, Santa Clara, CA, USA). Desorption step was performed in splitless mode (3 min) by setting the injector temperature at 260 °C. Chromatographic separation was performed with Rxi-1ms PDMS capillary column (54 m × 0.25 mm ID, 1 μm, Restek Corporation, Bellefonte, PA, USA) with helium as carrier gas at a constant flow rate of 1 mL/min. The GC oven temperature was programmed at 40 °C for 1 min, ramped 4 °C/min to 150 °C then at 8 °C/min to 250 °C held for 19 min. The detection was performed under electron impact ionization at 70 eV by operating in the full-scan acquisition mode with an m/z scanning range from 25 to 300. The transfer line was heated to 270 °C. The different compounds were identified using the NIST search engine and NIST mass spectra library.

### 2.8. Data Analysis

Given the complexity of the data array of the SPME-GC-MS measurements, the PARADISe method [18,19] was applied to obtain an efficient and rapid extraction of useful information, i.e., areas of deconvoluted peaks and their putative identification. PARADISe is a user-friendly software platform that employs PARAFAC2 (PARAllel FACtor analysis2) for peak deconvolution. PARADISe comes with a graphical user interface (GUI) and all necessary tools for GC-MS data processing, including (1) data visualization, (2) time-based data division, (3) PARAFAC2-based peak deconvolution, (4) validation and extraction of deconvoluted peaks, (5) compound identification using the NIST search engine and NIST mass spectra library or any other library in NIST format, and (6) the generation of a comprehensive metabolite table, including the area of every resolved peak. In this study, the defined intervals were 126, selected in order to enclose, when possible, only a single peak. Finally, NIST 08 was quested as library. The output matrix of PARADISe was analyzed through principal component analysis (PCA). PCA was performed on the NIR spectra and the UV–Vis lycopene spectra, too, to provide a comprehensive overview of the chemical characteristics of the samples throughout the considered storage time.

The pH and phenolic content were measured in triplicates, and the standard deviation was calculated accordingly.

One-way analysis of variance (ANOVA) was performed to assess whether there was a statistical difference among the investigated samples according to the pH and phenolic content.

### 2.9. Software

PARADISe approach was performed by PARADISe software version v.6.0.1. (http://www.models.life.ku.dk/paradise, accessed on 1 March 2023). PCA was carried out by using PLS_Toolbox 9.2.1 software (Eigenvector Research Inc., Manson, WA, USA) for MATLAB^®^. Experimental data were compared by applying analysis of variance (one-way ANOVA) by running in the Matlab^®^ 2023a environment (The Mathworks Inc., Natick, MA, USA). The level of significance was determined at *p* < 0.05 to see whether there were statistical differences between the mean values.

## 3. Results

### 3.1. pH and Total Phenolic Content

**Figure 1:**
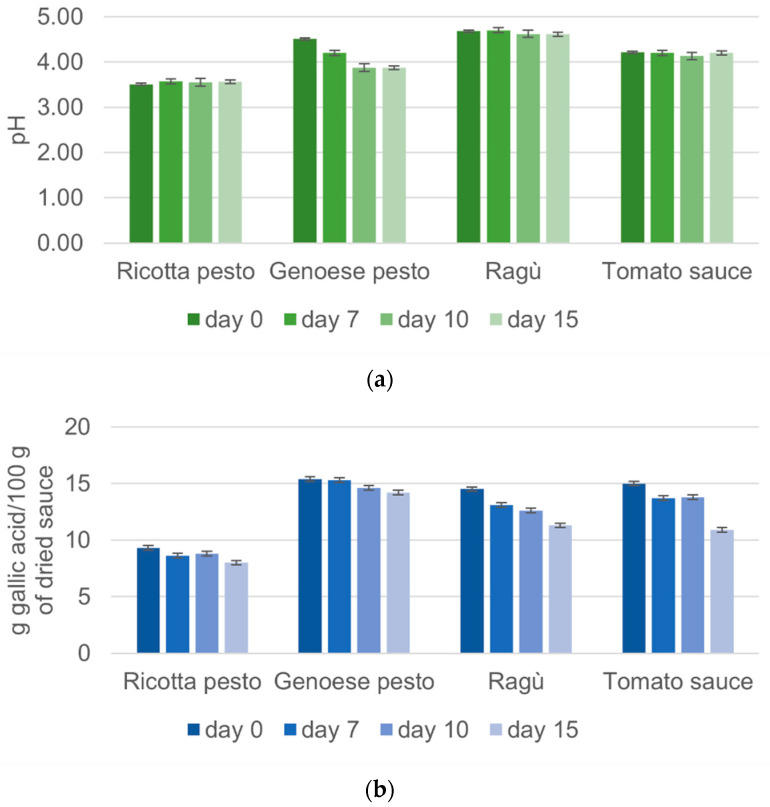
Variation of (**a**) pH values and (**b**) total phenolic content obtained for each investigated sample during the storage time.

In Figure 1a,b, pH and phenolic content values, respectively, for each investigated sample are shown as a function of the storage times.

Each waste sauce (hereafter indicated as “sauce”) was analyzed in triplicate and for both the measured parameters, the average values with associated standard deviation and *p*-values are reported in Table S1, Supplementary Materials.

As reported in Figure 1a, Genoese pesto initially shows a pH of 4.51, consistent with the fact that this pesto is generally stabilized by pasteurization and acidified with acidity regulators at pH < 4.6 to inhibit microbial development [24]. The pH values of Genoese pesto decreased by 14.2% during the 15 days of storage, probably due to the increase in heptanoic and octanoic acid concentration. These compounds are produced by oxidation reactions that occur over time, and they are among the mainly responsible molecules for the rise of rancidity and acidity, according to studies present in the literature on the shelf life of Genoese pesto [24,25]. Tomato sauce presents a pH mean value of 4.21, in agreement with the ECC 1986 Regulation, which states that tomato sauce must have a pH < 4.5 [26]. Tomato–meat sauces (like ragù) belong to the low acid foods category (pH > 4.6), according to many studies that report an initial pH of tomato and pork sauce between 4.6 and 5.0 [27]. The analyzed ragù sauce presents pH values (4.68) in line with the literature. In Figure 1a, it is possible to observe slight fluctuations in the pH values of both ragù and tomato sauce, consistent with findings reported in the previous literature [23,27]. Similar studies concerning ricotta pesto are not available. The present results revealed that ricotta pesto, characterized by the lowest pH values, exhibited remarkable stability with minimal variability during the storage time (the changes were not statistically significant, Table S1). This behavior suggests that the low pH values contribute to the pesto’s resilience against bacterial attacks, as well as to a stabilizing effect against ingredient decay.

The assessment of total phenolic content has been extensively explored in the literature, encompassing various components of tomatoes, including fruits and by-products such as seeds and peels [7]. Additionally, tomato-derived products like juice and ketchup have been scrutinized for their phenolic composition [28]. The phenolic content of tomato sauce quantified as 18.44 g/100 g of dried sauce (Figure 1b) closely mirrors findings in some tomato varieties, reporting 17 g/100 g of dried sauce [29]. This consistency is reasonable given that tomato sauce incorporates most parts of the tomato, predominantly the pulp, with a minor contribution from seeds and skins. Genoese pesto exhibits a high total phenolic content, approximately 19.02 g/100 g (Figure 1b), attributed to the rich polyphenol content in basil, the main ingredient. Conversely, ragù and ricotta pesto show lower values compared to tomato sauce, likely due to a lower tomato content in the recipe. These sauces also include ingredients like meat and cheese, which are not abundant in polyphenols. A noteworthy observation from Figure 1b is a clear decrease in total phenolic content over storage time for all investigated sauces. Phenolic compound degradation, likely due to peroxidase-mediated oxidation [28], results in the formation of quinones. The decrease in total phenolic content ranges from 21.40% to 25.40% for all sauces, except for ricotta pesto, which displays a lower decrease of 13.85%, maintaining minimal variability over time. Moreover, the results for tomato sauce align with previous studies in the literature [28,30], emphasizing the dynamic nature of phenolic content in different tomato-based products and their susceptibility to storage-induced changes. The changes in phenolic content in all the samples were statically significant (Table S1).

### 3.2. Qualitative Lycopene Analysis Results

**Figure 2:**
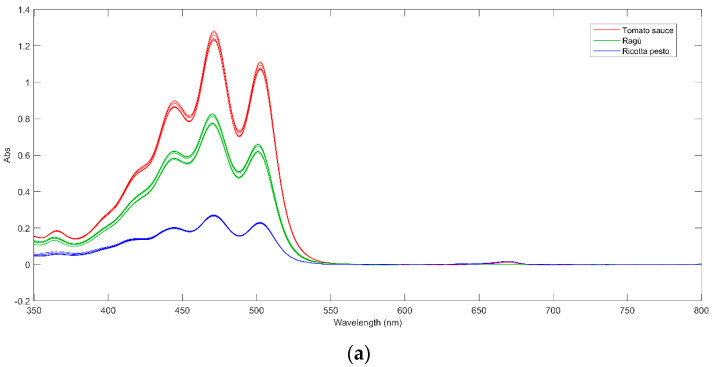

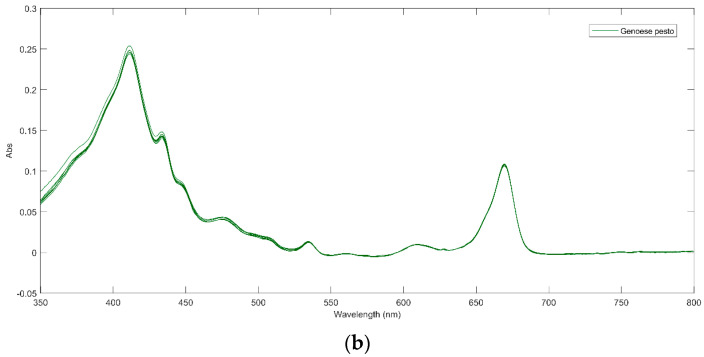
(**a**) UV–Vis spectra of tomato sauce (red line), ragù (green line), and ricotta pesto (blue line) at day 0; (**b**) UV–Vis spectrum of Genoese pesto at day 0.

The UV–Vis spectra of tomato sauce, ragù, and ricotta pesto, collected at day 0 are shown in Figure 2a. Each spectrum exhibits three main peaks at 440 nm, 475 nm, and 500 nm, accompanied by a shoulder around 420 nm and a low-intensity peak at 360 nm. Comparison with the standard lycopene spectrum [31] reveals a perfect overlap, confirming the presence of this compound in the samples. The tomato sauce (the red line in Figure 2a) exhibits the highest absorbance, indicative of the highest lycopene content. This aligns with the elevated tomato concentration in tomato sauce, specifically comprising 72% pulp and 4% concentrate, as outlined in Session 2.2. Ricotta pesto shows lower absorbance values, consistent with its lower tomato concentration, while ragù sauce obtains intermediate absorbance values.

Upon closer inspection of Figure 2a, it is possible to note that tomato sauce and ricotta pesto samples exhibit an additional low-intensity peak around 660 nm, probably due to chlorophylls and their derivatives (pheophytins) presence. These compounds are common green plant pigments, particularly abundant in basil, a key ingredient in these sauces. All these compounds show a very intense absorption between 420 and 470 nm and another intense band between 650 and 670 nm [7], which can be both found in the investigated Genoese pesto spectrum at day 0 (Figure 2b). Furthermore, from the analysis of Figure 2b, the presence of additional low-intensity peaks at 500, 540, and 610 nm leads to the conclusion that in Genoese pesto, the main pigment is pheophytin a, as reported by Masino et al., 2008 [32]. Considering the pasteurization of Genoese pesto, a common treatment for industrial milk-containing products, it is noted that this process quantitatively degrades chlorophylls into their derivatives (pheophytins) by substituting the Mg2^+^ ion with two hydrogen ions [33].

To gain information about the variability of the potential presence of lycopene in the samples over time, UV–Vis spectra acquired for the investigated samples were analyzed by means of principal components analysis (PCA). The UV–Vis spectra were organized into a bidimensional matrix with samples and replicates on the rows and UV–Vis variables on the columns. Genoese pesto signals were not considered in the matrix since they did not present the characteristic bands of lycopene. Before PCA analysis, all spectra were pre-processed using the automatic weighted least squares method, AWLS, to reduce the noise and the shift of baseline and mean centered.

PCA model was developed using two principal components, according to their explained variance (R2: 99.9%).

**Figure 3:**
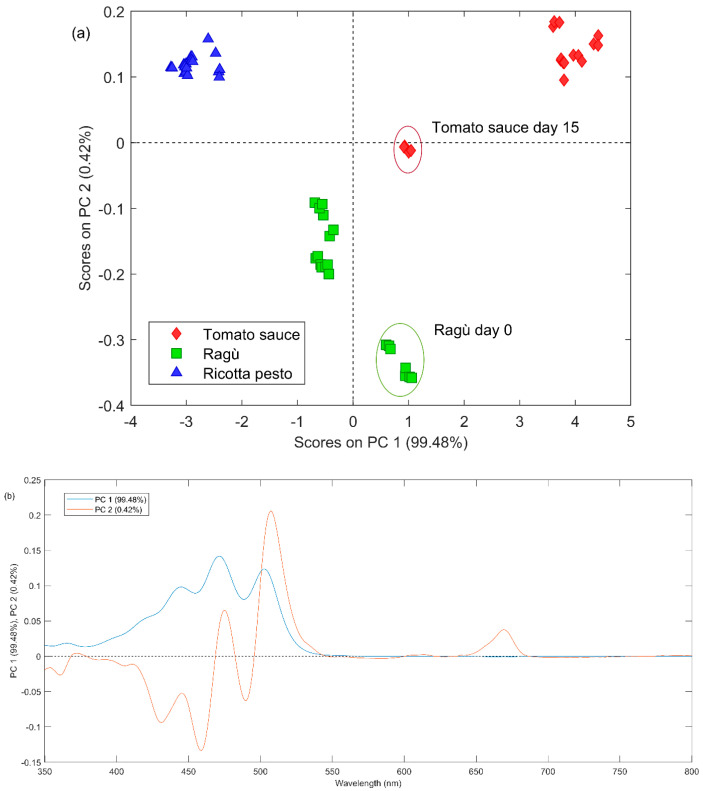
(**a**) PC1 vs. PC2 scores plot obtained by the PCA analysis of UV–Vis dataset; the red ellipse highlights tomato samples at day 15 and the green one highlights ragù samples at day 0. (**b**) Loading plot of PC1 (blue line) and PC2 (red line) of PCA applied on UV–Vis dataset.

In Figure 3a, the PC1 vs. PC2 scores plot is reported, representing the different types of investigated sauces with different symbols and colors. In the first principal component (PC1), the most significant differences emerge between ricotta pesto (negative PC1 scores) and tomato-based sauce (positive PC1 scores). Ragù spectra are separated from the other two types mainly on the second component (PC2) with negative scores. From the PC1 loadings figure (Figure 3b), all the lycopene characteristic bands present positive values, therefore, from a synergistic comparison with the scores plot, it is possible to state that tomato-containing samples seem to have a higher lycopene content with respect to the ricotta pesto ones. On the second principal component, ragù samples generally present a higher absorbance from 350 nm to 460 nm (with negative PC2 loadings values) and lower from 470 nm to 550 nm (with positive PC2 loadings value, Figure 3b). Regarding the variability of lycopene content as a function of the storage time, from a comprehensive analysis of Figure 3a, some differences emerge only for tomato and ragù samples. Tomato sauce exhibits, for over 10 days, the highest lycopene content among all samples. At day 15, the lycopene amount seems to decrease, and it becomes similar to the ragù content. Lycopene is very sensitive to oxygen, light, and heating; therefore, it could be easily degraded during tomato product storage. Lycopene is an efficient antioxidant that quenches highly reactive singlet oxygen and traps peroxyl radicals (ROO·), forming peroxyl radicals capable of acting as a prooxidant and undergoing autoxidation. Oxidative degradation happens by introducing an oxygen function by substitution of a methyl or methylene group or addition to a carbon-carbon double bond, with scission of the carbon chain [34]. Ragù samples have positive PC1 values at day 0, while negative PC1 values for the other days. In this case, lycopene could immediately undergo degradation and then remain almost constant in terms of concentration for the next 15 days.

### 3.3. NIR Spectra Analysis

**Figure 4:**
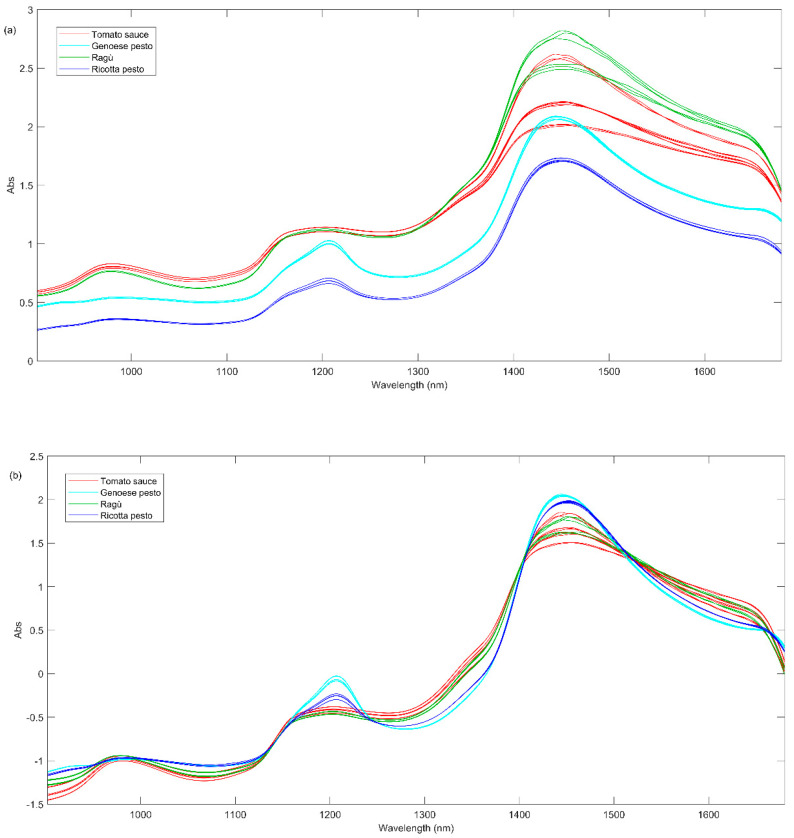
(**a**) Raw NIR spectra of all sauces at day 0; (**b**) NIR spectra at day 0 after SNV pretreatment.

The NIR spectra acquired for samples at day 0 are graphically shown in Figure 4a and they seem to be quite peculiar for some sauces.

From the graphical inspection of the figure, the absorption band at 1160–1200 nm, appearing in almost all NIR spectra (Figure 4a), could be related to the overtones of –C–H stretching from alkenes or polyenes (1170–1180 nm), C=O carbonyl stretching from aliphatic hydrocarbons (1160–1170 nm) and C–H stretching from aromatic hydrocarbons (1142–1152 nm). These compounds can also present a combination of three bands at 1365, 1422, and 1451 nm. The band at about 1210 nm is symptomatic of C-H methylene stretching overtone from aliphatic hydrocarbons (1211–1222 nm). The broad band between 1400 and 1500 nm could be related to the following different vibration overtones: (i) C–H combination from aromatic hydrocarbons (1422–1451 nm); (ii) O–H stretching from water or phenols (1421 and 1469 nm), secondary and tertiary alcohols (1469–1473 nm) and diols (1420 and 1465 nm) and polyphenols; (iii) N-H from amides/proteins (1468–1485 nm) [35].

In order to obtain more information about the similarities or differences among all the investigated sauces, PCA analysis was carried out on the whole investigated NIR signals acquired for samples at different times of storage. Before PCA, the spectral data were pre-treated using a standard normal variate, SNV, and baseline correction (Figure 4b).

**Figure 5:**
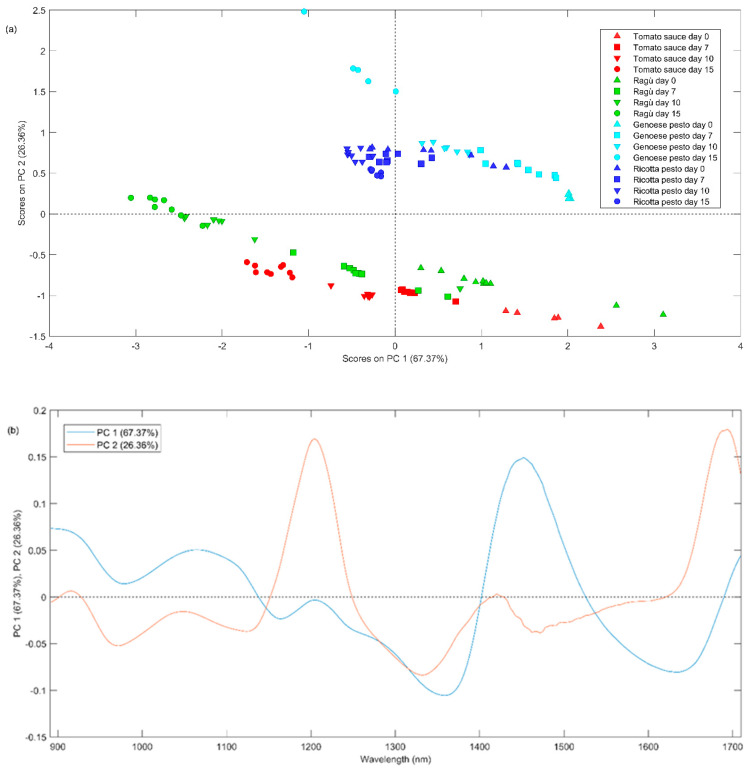
(**a**) Scores plot and (**b**) loadings plot of PC1 vs. PC2 of PCA applied on NIR dataset for days 0, 7, 10, and 15.

Finally, the spectra acquired for all the samples were mean-centered and the PCA model was built considering two principal components explaining the 93.73% of the total variance. In Figure 5a, the PC1 vs. PC2 scores plot is reported, representing the different types of investigated sauces with distinct colors and different times of storage with distinct symbols. From Figure 5a, it can be seen a lower repeatability among some replicates (same symbols and same colors) due to a higher heterogeneity of these samples.

The first principal component seems to describe the effect of time, with the samples measured on day 0 (triangle up symbols) having positive values, while those measured on days 7 (square symbols), 10 (triangle down symbols), and 15 (circle symbols) have progressively lower values. It is noteworthy that the most significant shift occurs during the period between day 7 to day 10. The second principal component differentiates samples based on their composition, with negative values for tomato-based sauces and positive values for Genoese pesto. From the PC1 loadings plot (blue line, Figure 5b), the band at 1470 nm presents positive values; therefore, it is possible to state that this band could distinguish samples based on storage time. As described before, this peak could be attributed to O-H stretching from polyphenols. A plausible hypothesis is that a decrease in PC1 score values over time may be correlated with a reduction in polyphenol content, in agreement with the observations highlighted in Session 3.1. Pesto Genoese samples present a more intense band at 1200 and 1700 nm (positive PC2 loadings), which can be associated with C-H methylene stretching overtones from aliphatic hydrocarbons.

### 3.4. Putatively Identification of the Volatile Compounds by SPME-GC-MS

The volatile compounds (VOCs) of the different samples were identified by matching sample mass spectra with those of the National Institute of Standards and Technology (NIST) mass spectral library. A volatile compound was considered “identified” if its mass spectral fit value was 85 (default value) or more. The identified compounds, together with their retention time, Rt, and match factor, are reported in Table 1.

First, the analyte areas were divided by the internal standard areas. In particular, the analytes were divided into two groups: those with retention time, Rt, values less than 14 min, and those with Rt greater than 14 min. At this point, the areas of the analytes in the first group were divided by the area of the 4-methyl-2-pentanol standard, while the areas of the analytes in the second group were divided by the area of the 2-phenylacetate standard. Based on Table 1, this choice is dictated by the closeness in the chromatogram in terms of the Rt of the analytes to one standard rather than the other. In this way, relative concentrations were obtained, and they were used for PCA.

The volatile profile of pasta condiment sauce is characterized by a complex pattern of volatile compounds. These molecules belong to different chemical classes, namely carboxylic acids (Rt: 23.37–55.59 min), alcohols (Rt: 13.98–32.55 min), aldehydes (Rt: 20.22–36.71 min), ketones (Rt: 23.99–32.96 min), esters (Rt: 33.95), terpenes (Rt: 22.94–39.92), etc. The presence of some of these compounds could be an indicator of the quality of these wastes, as they could be directly linked to the presence of basil, olive oil, and tomato. Indeed, some volatile compounds, such as 3-methylbutanal, 6-methyl-5-epten-2-one, and 1-hexanol are linked to tomato; other molecules, i.e., terpenes, result from the presence of basil [36], walnuts, and tomato too [37,38,39]. In particular, terpenes (Rt: 22.94–39.92) are the predominant substances of the aroma composition of the investigated samples. These compounds constitute the primary bioactive components found in plant essential oils. Their molecular structures consist of carbon backbones composed of isoprene units, which can be reorganized into cyclic structures [37]. Monoterpenes, formed by two isoprene units, are the most common terpenes and, therefore, mainly responsible for the aroma profile, followed by sesquiterpenes [38]. Diterpenes, triterpenes, and tetraterpenes with their oxygenated derivatives (terpenoids) are also detected in small amounts in different studies present in the literature [39]. α-pinene, camphene, limonene, eucalyptol, linalool, neral, geranial, eugenol, and methyl eugenol are terpenes that contribute to the aroma of tomatoes, which can also be clearly detected in their processed products. All these molecules, except for neral and geranial, constitute the basil aroma [36] together with 2-β-pinene, myrcene, γ-terpinene, p-cymene, sabinene, and δ-3-carene, and they are found in the volatile fraction of Genoese pesto [40].

The presence of heptane (Rt: 12.41 min), (Z)-3-hexen-1-ol acetate (Rt: 24.94), nonanal (Rt: 29.19 min), n-decanal (Rt: 32.64 min), octanoic acid (Rt: 31.20 min), decanoic acid (Rt: 36.48 min) and myristicin (Rt:39.82 min) could be owing to the addition of olive and sun-flower oils to the sauce [41].

**Figure 6:**
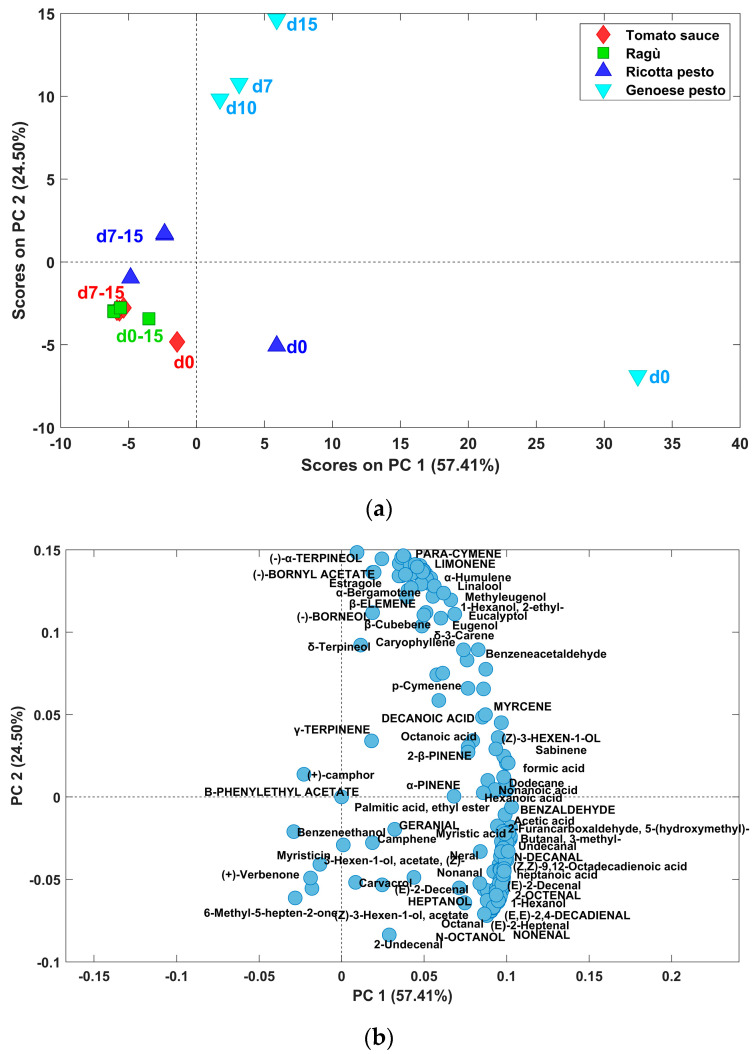
Scores (**a**) and loadings (**b**) plot of PCA model built on volatile compounds areas.

Tomato sauce aroma was also due to the presence of (Z)-3-hexen-1-ol (Rt: 18.31 min), 1-hexanol (Rt:18.87 min), 6-methyl-5-hepten-2-one (Rt:23.99 min) and hexanoic acid (Rt: 23.37 min), (E,E)-2,4-decadienal (Rt: 35.53 min), benzaldehyde (Rt: 22.82 min), 2-undecenal (Rt: 36.72 min), 2-octenal (Rt:27.10 min), dodecane (Rt:32.99 min) and 5-(hydroxymethyl)-2-furancarboxaldehyde (Rt: 32.30 min), consistent with many studies present in the literature [37,38,39]. Genoese pesto volatile fraction resulted composed mainly by 3-methylbutanal (Rt: 9.39 min), heptane, octane (Rt:16.57 min), (Z)-3-hexen-1-olo, (Z)-3-hexen-1-ol acetate, 1-hexanol, 1,3-dimethylbenzene (Rt: 19.39 min), heptanal (Rt: 20.23 min), (E)-2-heptenal (Rt: 22.66 min), octanal (Rt: 24.81 min), n-octanol (Rt: 27.79 min), nonanal, heptanoic acid (Rt: 27.46 min), octanoid acid, n-decanal, undecanal (Rt: 35.39 min), 2-undecenal, in accordance with different studies [40,41]. Many of these compounds are characteristic of nuts as well. As regards ragù sauce, only one compound, heptanol, can be attributable exclusively to the meat presence (https://foodb.ca/, accessed on 19 July 2024), while many others are released from the cheeses in the sauces (3-methylbutanal, hexanoic acid, octanal, nonanal, octanoic acid, (E)-2-decenal), according to Salvadeo et al. [41]. The area values of the 160 compounds resolved by the PARADISe approach (Table 1) for each investigated sauce at different storage times were organized in a bidimensional matrix, and, after autoscaling pretreatment, PCA was carried out considering two principal components (R2: 81.91%). The scores plot, reported in Figure 6a, reveals significant variations mainly attributed to Genoese pesto sauces (blue symbols in the figure), with the highest PC1 scores for the initial sample (day 0) and the highest PC2 scores for the other ones.

The loadings plot, in Figure 6b, shows that these samples are characterized by higher area values for a broad spectrum of volatile compounds. Furthermore, the distribution trends in scores among five types of sauces highlight distinct patterns, with Genoese pesto and ricotta pesto samples at day zero (positive PC1 scores) with a more intensive VOC profile. In addition, for each type of sample, those at time zero display higher scores in PC1 compared to those stored at 7, 10, and 15 days. Moreover, the latter group appears to have a stable aromatic composition, as most samples cluster closely together. This suggests that when samples are stored at 4 °C, their volatile composition undergoes an important change from 0 to 7 days of storage, after that, it remains relatively stable. From the PC1 vs. PC2 loadings plot in Figure 6b, it is evident that samples at day 0, located on the positive side of PC1 scores, are characterized by a higher content of almost all volatile compounds.

## 4. Conclusions

The results of the study underline the importance of a comprehensive understanding of the chemical composition of this type of waste. The presence of bioactive compounds adds value to the by-products and underlines their potential as a valuable resource that, if properly managed, can contribute to sustainable and environmentally friendly practices in the industry. The main findings are summarized below, on their basis, future research is worth aiming at the development of environmentally sustainable analytical methodologies for extracting the bioactive compounds that emerged from this research.

*(a) Assessment of chemical profile of the four investigated pasta sauce wastes*

Overall, the obtained results revealed that the investigated samples are effectively rich in bioactive compounds, such as lycopene and polyphenols, supporting the idea that the by-products of the pasta sauce industry contain valuable substances.

However, it is crucial to acknowledge that this represents only a preliminary exploration of the potential bio-activity compounds within these sources. Given the complexity of food matrices and the diverse range of compounds that could be present, it is imperative to recognize the necessity for further investigations for the determination of other high-value compounds such as other carotenoids, fatty acids, etc.

Comparing the different sauces that were characterized, tomato sauce and ragù were the most similar ones, sharing a high tomato juice content and many of the other ingredients. This consideration is mainly based on the results obtained by PCA analysis of the NIR spectra (Section 3.3), as well as by comparison of their pH and total phenolic content values. Genoese pesto exhibited unique properties due to the absence of tomato (e.g., no lycopene content), while ricotta pesto resulted in having properties somewhere between Genoese pesto and tomato-based sauces. The differences observed in the volatile fraction profile (PCA of resolved peaks area, Figure 6) highlight that Genoese pesto and ricotta sauces are generally richer in volatile organic compounds than tomato and ragu-based ones.

Albeit preliminary, these results furnish a first indication of which bioactive compounds are present and in which sauces are more abundant, giving some directions on where to put forthcoming research to pursue valorization.

*(b) Chemical profile variation with storage time*

Firstly, it has been observed that all the sauces, when stored in the fridge at 4 °C after opening, have shown a decrease in the concentration of most of the analytes, such as lycopene, polyphenols, and most of the aromatic compounds responsible for the aromatic profile. The most important changes in composition took place from 0 to 7 days of storage, after that time, the products remained relatively stable. By looking at the NIR spectra, the entity of the variation seems to be similar for the four sauces by-products investigated, while limiting to the volatile fraction, the impact is clearly higher for Genoese pesto. From PCA analysis of the UV–Vis spectra, it can be observed that lycopene decrease is higher in tomato sauce by-product. These findings provide crucial insights into the potential variability and stability of bioactive compounds in pasta condiment waste and could aid proper planning of waste management from a valorization perspective. Indeed, this aspect is very important from an industrial perspective, where waste is stored for days before being reused or discarded.

Finally, considering all the investigated variables, it is important to recognize that this study has several limitations that may affect the generalization of the results. Several key issues deserve attention. In this study, a relative quantitative assessment using the ratio of the peak areas of resolved compounds has been carried out. When using HS-SPME-GC/MS analysis, quantification is particularly critical because of the several factors that may influence the accuracy of the results, such as the matrix effect, due to the highly complex and heterogeneous composition of the analyzed waste, which can influence not only the chromatographic step but also the headspace-extracting phase partition coefficient. Therefore, an accurate quantification considering all these reasons could be performed in further studies focusing only on a few analytes, e.g., the most valuable, most abundant, etc. Furthermore, this study did not include the quantification of bioactive compounds such as lycopene, which is crucial to demonstrate the potential valorization of waste materials. Lycopene is known for its health benefits, but it has significant stability problems [42,43]. After this first insight and having assessed its presence, accurate quantification of lycopene could be considered in further studies, where it will be possible to extend the sampling campaign.

## Figures and Tables

**Table 1:** Putatively identified analytes, their retention times, and the match factor.

Compound Name	Match Factor	Retention Time (min)
Acetic acid	96	8.87
3-methyl-Butanal	90	9.39
Heptane	88	12.41
4-methyl-2-Pentanol	92	13.98
Octane	94	16.57
(Z)-3-Hexen-1-ol	96	18.31
1-Hexanol	87	18.87
1,3-dimethyl-benzene	96	19.40
Heptanal	90	20.23
Nonane	86	21.14
(E)-2-Heptenal	93	22.66
Benzaldehyde	87	22.83
α-Pinene	95	22.94
Hexanoic acid	97	23.37
Heptanol	96	23.50
Camphene	95	23.64
6-Methyl-5-hepten-2-one	92	23.99
Sabinene	93	24.45
Myrcene	97	24.88
2-β-Pinene	91	24.81
(Z)-3-Hexen-1-ol acetate	85	24.94
Octanal	93	24.81
2-ethyl-1-hexanol,	85	26.15
Benzeneacetaldehyde	87	26.29
P-Cymene	93	26.42
Limonene	95	26.86
Eucalyptol	98	26.91
2-Octenal	93	27.10
δ-3-Carene	96	27.24
Heptanoic acid	87	27.46
n-Octanol	92	27.80
γ-Terpinene	93	27.97
Nonanal	96	29.19
Linalool	94	29.22
Benzeneethanol	93	29.44
p-Cymenene	86	28.98
p-Mentha-1,5,8-triene	95	30.02
Nonenal	96	31.10
(+)-camphor	94	31.12
Octanoic acid	85	31.20
(−)-Borneol	92	31.99
3-Pinanone	86	32.07
5-(hydroxymethyl)-2-Furancarboxaldehyde	96	32.30
δ-Terpineol	87	32.26
Estragole	93	32.53
(−)-α-Terpineol	93	32.55
n-Decanal	86	32.64
(+)-Verbenone	96	32.97
Dodecane	86	33.00
Neral	88	33.64
2-Phenylethyl acetate	96	33.95
Nonanoic acid	96	34.08
(E)-2-Decenal	97	34.19
Geranial	85	34.35
(E,E)-2,4-Decadienal	86	35.03
Carvacrol	95	35.14
(−)-Bornyl acetate	96	35.27
Undecanal	86	35.39
Triacetin	85	35.53
Decanoic acid	87	36.48
Eugenol	98	36.56
2-Undecenal	96	36.72
Methyleugenol	85	37.31
β-Elemene	93	38.01
Caryophyllene	96	38.85
α-Bergamotene	96	38.85
α-Humulene	90	39.49
(E)-β-Farnesene	88	39.79
Myristicin	96	39.83
β-Cubebene	85	39.92
Lauric acid	86	40.37
Myristic acid	86	43.84
Palmitic acid	96	48.48
Palmitic acid, ethyl ester	86	49.64
(Z,Z)-9,12-Octadecadienoic acid	85	54.43
cis-13-Octadecenoic acid	96	54.74
Stearic acid	86	55.59
Oleic acid, ethyl ester	95	56.19

## Data Availability

The raw data supporting the conclusions of this article will be made available by the authors on request.

## Supplementary Materials

The following supporting information can be downloaded at: https://www.mdpi.com/article/10.3390/foods13183018/s1, Table S1: Total phenolic content and pH measured at different time with the associated standard deviation.
