# Supplementary material for: Chemical Characterization and Temporal Variability of Pasta Condiment By-Products for Sustainable Waste Management
Source: Foods. 2024 Sep 23;13(18):3018. doi: 10.3390/foods13183018 (PMC11431017; doi:10.3390/foods13183018)
Supplement: Supplementary file 1 [file foods-13-03018-s001.zip › foods-3202895-supplementary.pdf]

Supplementary materials

Table S1. Total phenolic content and pH measured at different time with the associated standard deviation.

	Total phenolic content (g gallic acid/100 g of dried sauce)					pH				
	day 0	day 7	day 10	day 15	*p*-value	day 0	day 7	day 10	day 15	*p*-value
Ricotta pesto	9.3 ± 0.2	8.6 ± 0.2	8.8 ± 0.2	8.0 ± 0.2	<0.05	3.51 ± 0.03	3.57 ± 0.03	3.55 ± 0.03	3.56 ± 0.03	0.6
Genoese pesto	15.4 ± 0.2	15.3 ± 0.2	14.6 ± 0.2	14.2 ± 0.2	<0.05	4.51 ± 0.02	4.20 ± 0.02	3.87 ± 0.02	3.87 ± 0.02	<0.05
Ragù	14.5 ± 0.2	13.1 ± 0.2	12.6 ± 0.2	11.3 ± 0.2	<0.05	4.68 ± 0.01	4.70 ± 0.01	4.62 ± 0.01	4.61 ± 0.01	<0.05
Tomato sauce	15.0 ± 0.2	13.7 ± 0.2	13.8 ± 0.2	10.9 ± 0.2	<0.05	4.21 ± 0.01	4.20 ± 0.01	4.13 ± 0.01	4.20 ± 0.01	<0.05
